# A mixed methods study to evaluate the impact of a student-run clinic on undergraduate medical education

**DOI:** 10.1186/s12909-021-02621-y

**Published:** 2021-03-25

**Authors:** Nathan G. Rockey, Taylor M. Weiskittel, Katharine E. Linder, Jennifer L. Ridgeway, Mark L. Wieland

**Affiliations:** 1 Mayo Clinic Alix School of Medicine, 200 First Street SW, Rochester, MN 55905 USA; 2 Mayo Clinic Graduate School of Biomedical Sciences, 200 First Street SW, Rochester, MN 55905 USA; 3 Center for the Science of Healthcare Delivery, Mayo Clinic, 200 First Street SW, Rochester, MN 55905 USA; 4 Division of Community Internal Medicine, Mayo Clinic, 200 First Street SW, Rochester, MN 55905 USA

**Keywords:** Undergraduate medical education, Student-run clinics

## Abstract

**Background:**

The purpose of this study was to evaluate the extent to which a longitudinal student-run clinic (SRC) is meeting its stated learning objectives, including providing critical community services and developing physicians who more fully appreciate the social factors affecting their patients’ health.

**Methods:**

This was a mixed methods program evaluation of an SRC at Mayo Clinic Alix School of Medicine (MCASOM). A survey was conducted of medical students who had participated in the clinic and seven interviews and three focus groups were conducted with SRC patients, students, faculty, staff, and board members. Transcripts were coded for systematic themes and sub-themes. Major themes were reported. Survey and interview data were integrated by comparing findings and discussing areas of convergence or divergence in order to more fully understand program success and potential areas for improvement.

**Results:**

Greater than 85% of student survey respondents (*N* = 90) agreed or strongly agreed that the SRC met each of its objectives: to provide a vital community service, to explore social determinants of health (SDH), to understand barriers to healthcare access and to practice patience-centered examination. Qualitative data revealed that the SRC contextualized authentic patient care experiences early in students’ medical school careers, but the depth of learning was variable between students. Furthermore, exposure to SDH through the program did not necessarily translate to student understanding of the impact of these social factors on patient’s health nor did it clearly influence students’ future practice goals.

**Conclusions:**

The MCASOM SRC experience met core learning objectives, but opportunities to improve long-term impact on students were identified. Participation in the SRC enabled students to engage in patient care early in training that is representative of future practices. SRCs are an avenue by which students can gain exposure to real-world applications of SDH and barriers to healthcare access, but additional focus on faculty development and intentional reflection may be needed to translate this exposure to actionable student understanding of social factors that impact patient care.

**Supplementary Information:**

The online version contains supplementary material available at 10.1186/s12909-021-02621-y.

## Background

Student-run clinics (SRCs) have become an important component of the early clinical curriculum at many medical schools in the United States [1, 2]. SRC experiences typically occur in the pre-clinical years with varying involvement of upper-class medical students, and they address the “service learning/community service” competency of the Liaison Committee on Medical Education 2020 Standards for Accreditation of Medical Education Programs (section 6.6) [3].

Through participation in SRCs, students may learn responsibility for patient care early in their education, [4] increase their systems-based knowledge (i.e., understanding of issues in the larger context of health care), [5, 6] and understand social determinants of health (SDH) and social medicine more broadly. Prior studies among students (using surveys, qualitative interviews, and mixed methods approaches) have shown that they enjoy the SRC experience [7, 8], find it to be a beneficial educational experience for primary care topics [9], and perceive that exposure to SDH through an SRC is important to develop an understanding of barriers their patients face [6]. Furthermore, inter-professional education in SRCs has been shown to be a positive aspect of these clinic experiences [10, 11]. From a patient perspective, assessment of patient satisfaction with SRCs has also generally been reported as high [12–14], although satisfaction with wait-times and perceived privacy have been found to be lower in the SRC setting [12].

The Mayo Clinic Alix School of Medicine (MCASOM) SRC is a required longitudinal component of the second year clinical education for all medical students.

The stated objectives of the SRC are:

1. To provide a vital community service
2. To explore the social determinants of health through the lives and circumstances of MCASOM SRC patients
3. To understand barriers to healthcare access through experiences of MCASOM SRC patients
4. To practice patient-centered medical evaluation and examination

The objective of this study was to evaluate the extent to which a longitudinal student-run clinic (SRC) was meeting its stated objectives, including providing critical community services and developing physicians who more fully understand the lives of patients impacted by health disparities. Unlike prior research that has often focused on single stakeholder groups like patients [14] or students [6], we sought the perspectives of multiple stakeholders. This study also extends prior literature by using mixed methods to elucidate not only *whether* the program was satisfactory and increased system knowledge but *how or under what circumstances* students achieved complex objectives like exploring SDH and practicing patient-centered examination in a population vulnerable to health disparities and limited access to care. These findings can be used to evaluate whether the program is meeting its objectives and to inform improvements or identify lessons learned for the field of medical school SRC programs.

## Methods

This evaluation employed a convergent mixed methods design where surveys and interviews/focus groups were conducted concurrently and the results were compared after the initial quantitative and qualitative analysis was complete. The use of multiple data sources and types of data (i.e., triangulation) was meant to provide a fuller understanding of whether the program was effective [15, 16]. Survey data provided assessment of how well students thought the SRC met stated objectives; while individual interviews and focus groups provided an opportunity to explore the perspectives of several types of stakeholders in greater depth and understand how the program met these objectives or what gaps may still persist.

### Setting

The MCASOM SRC was founded in 2009 as a subset of the Rochester Area Salvation Army Good Samaritan Health Clinic, and serves as an important source of healthcare for uninsured and underinsured patients in Olmsted County, Minnesota and adjacent counties [17]. The clinic operates two half-days per week; each session is staffed by 8 medical students, 2 faculty members, a pharmacist, pharmacy students, and a receptionist. The clinic is further supported by an SRC program manager and a nurse manager who oversees the broader free clinic. The initial history and physical examination are performed by a medical student, who then presents the patient to a physician preceptor. The preceptor sees the patient along with the student and prescribes medications, provides referrals to other services offered at the clinic (e.g. psychiatry clinic, eye clinic), and offers a follow-up appointment if indicated. Additionally, patients may be referred for laboratory testing or for a visit at Mayo Clinic if there is need for a complex workup.

### Recruitment

Email invitations were sent to all second year students, SRC student leaders, and physicians who staff the clinic, inviting them to participate in a focus group. Key stakeholders were identified by the study team and were invited via email to participate in an individual interview. Patients who were familiar with the SRC were identified by the staff and were invited to participate during normal SRC hours after their visit. This study was deemed exempt by the Mayo Clinic IRB (IRB **ID:** 18–009983).

### Quantitative data collection

Patient demographics for the 2018–19 academic year were abstracted from the SRC medical record. We administered an online survey delivered by school email in March, 2019 to all MCASOM students who had participated in the SRC: current second, third and fourth year students. Two follow up emails were sent to increase participation rate (2 weeks and 3 weeks after initial email). This survey assessed the degree to which students believed the MCASOM SRC was meeting its four learning objectives. The complete survey is available as Additional File 1.

### Qualitative data collection

We developed interview and focus group guides based on the program’s learning objectives, with questions on constructs similar to those in the survey. Questions also explored participants’ own understanding of the program’s goals, what factors are related to success, and what challenges remain. Full interview and focus group guides are available as Additional File 2. All interviews and focus groups were conducted by members of the study team with training in qualitative moderation (K.L., T.W., and N.R). Participants provided oral informed consent prior to the interview/focus group. The interviews and focus groups were recorded, transcribed, and de-identified for analysis.

### Data analysis

Quantitative data were reported with descriptive statistics. Qualitative data were analyzed using thematic analysis methods [18]. First, members of the study team read each transcript, wrote initial impressions of the data, and met to discuss their impressions. They subsequently developed a codebook with a priori topics from a review of the literature as well as the preliminary reading of the data. Transcripts were then systematically coded by three members of the study team using this framework. Disagreements about coding excerpts were resolved by consensus. Coded data were reviewed and discussed by the study team in order to identify overarching themes related to the study aim. Development of themes was initially based on the four SRC learning objectives and then expanded to include themes that arose naturally from the data. Quotes presented in this text have been edited for grammar and clarity.

### Data integration

Qualitative and quantitative data were initially analyzed separately and then major themes in the qualitative findings were considered alongside the survey findings. Specifically, the study team compared survey and qualitative findings, discussed areas of convergence/divergence, and considered how stakeholder perspectives in the qualitative data—including those of non-students—expanded what the student surveys showed.

## Results

### Patient demographics

In the 2018–19 academic year, 437 unique patients were seen at the SRC. Patient demographics are shown in Table 1. The majority of MCASOM SRC patients were Hispanic (38%) or Caucasian (28%), and 70% lived below 133% of the federal poverty level. When asked what they would do without the SRC, 56% indicated they would go without medications and 29% did not know what they would do. A majority (58%) of MCASOM SRC patients were ineligible for health insurance, mostly due to undocumented immigration status. The second largest category (31%) of MCASOM SRC patients were waiting for an insurance application to be processed, new insurance to start, or an enrollment period to open. The remaining patients (11%) reported underinsurance or cost barriers to adequate insurance. MCASOM SRC provides free prescription medications, and 70% of MCASOM SRC patients received at least one prescription during their visit.

**Table 1:** Demographics of patients of the MCASOM SRC in 2019

Insurance Status	
Ineligible^1^	262
Insurance Pending^2^	84
Insurant enrollment information provided^3^	71
Underinsured or too expensive^4^	20
**Ethnicity**	
Hispanic	168
Caucasian	123
African American	87
Asian or Pacific Islander	58
Native American	1
**Age**	
35–54 Years	210
55–64 Years	100
65+ Years	56
25–34 Years	41
18–24 Years	22
13–17 Years	2
7–12 Years	3
0–7 Years	3
**Income Level**	
Under 133% of the poverty level (eligible for MA)	307
Between 133 and 200% (eligible for MN Care)	50
Between 200 and 400% (eligible for open market)	80
**What would Patients do without The Salvation Army?**	
Go without medications	246
Doesn’t know	126
Find somewhere else	31
Nothing	16
Go to the Emergency Room	18
**Total**	**437**

^1^: Uninsured due to immigration status (225) or income level that is too high for public insurance (37)

^2^: Uninsured and in various stages of insurance application process.

^3^: Uninsured and given information about public insurance.

^4^: Enrolled in Medical Assistance program or Medicare [12], or have high deductibles [8]

### Student survey results

**Fig. 1:**
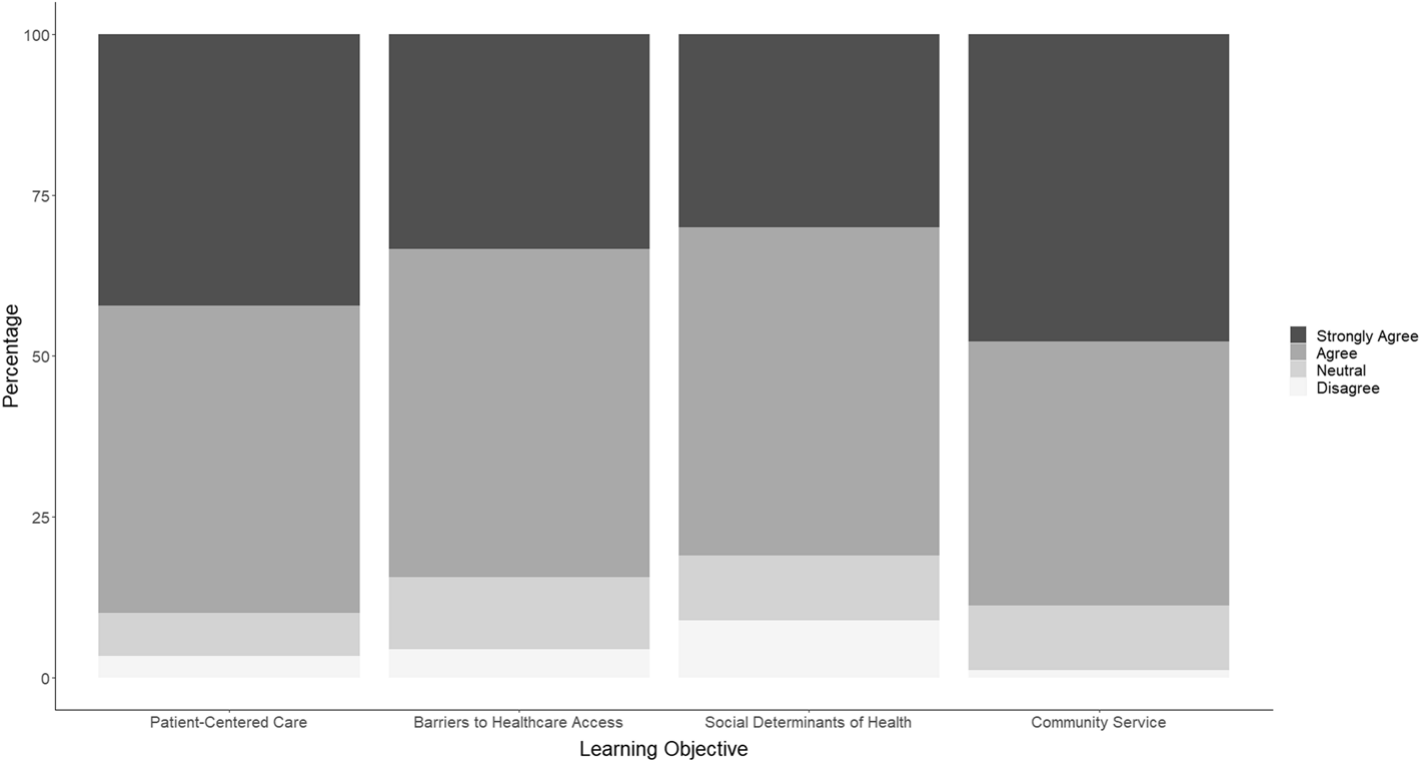
MCASOM Student Survey Responses. MCASOM student survey responses (*n* = 90) on the extent to which the SRC is meeting the learning objectives: (1) To provide a vital community service (2) To explore the social determinants of health through the lives and circumstances of MCASOM SRC patients (3) To understand barriers to healthcare access through experiences of MCASOM SRC patients (4) To practice patient-centered medical evaluation and examination

A total of 90 students completed the survey (response rate approximately 60%). Most students (89%; *n* = 80) agreed or strongly agreed that MCASOM SRC provided a vital community service. Eighty four percent (*n* = 76) of students “agreed” or “strongly agreed” that MCASOM SRC facilitated learning about barriers to healthcare access. Eighty one percent (*n* = 73) of students agreed or strongly agreed that MCASOM SRC allowed students to explore and better understand SDH. Finally, 90% of students (*n* = 81) agreed or strongly agreed that MCASOM SRC serves as an opportunity to practice patient centered care (Fig. 1). Responses did not differ significantly by year of training or anticipated specialty choice (data not shown).

### Qualitative results

Eight interviews were conducted with key stakeholders including board members of the broader free clinic (2), current patients (3), the SRC medical director (1), the SRC program manager (1), and the SRC pharmacist (1). In addition, three focus groups were conducted: one with SRC faculty members, one with SRC student participants, and one with SRC student leaders. Qualitative themes were reported by overarching categories that represented the SRC program objectives. The first category included themes related to the practice of healthcare and the learning of that practice. The second focuses on learning as an opportunity to understand the patient experience (Table 2).

**Table 2:** Qualitative themes by category

Category	Themes
1. SRCs as a vital community service and opportunity to learn patient-centered practices	• Realistic patient care experience • Self-directed learning and leadership opportunities
2. SRCs as an opportunity to understand how patients experience health and care	• Exposure to social determinants of health and barriers to healthcare access • Patients as teachers • Effect on future practice

### SRCs as a vital community service and opportunity to learn patient-centered practices

#### Realistic patient care experience

During a clinic morning, students must perform a focused history and physical exam, present the patient to the preceptor and write a note. Students felt that this provided a realistic clinical experience in terms of time constraints, note writing, and inter-professional team dynamics. The time constraints in particular were juxtaposed against other experiences in the pre-clinical years and were cited as providing preparatory value for the clinical years:

“I think the most beneficial thing in this was just literally the time constraints themselves and just how realistic that’s going to be to some of our actual practices, and you won’t have all night to write a note and look up everything you want.” (student)

MCASOM SRC is also staffed by pharmacy students from associated schools who are present most clinic days to assist in managing patient medication regimen and providing patient education. This unique aspect of MCASOM SRC was cited as a positive learning experience:

“I think it’s a great opportunity to learn from one another and value each other’s professions and what your specialties are, and I guess in my mind I’d like to believe that seeing how interdependent the two professions are and just that opportunity to learn and share what you’ve learned” (lead pharmacist)

The interdisciplinary nature of MCASOM SRC thus provides a realistic environment for future careers working in a multi-professional health care team.

#### Self-directed learning and leadership opportunities

Students described how the SRC relies on self-directed learning and that there is a wide range of student commitment to the process. Students in the focus group understood that this has implications for patient-centered care and for the depth of learning that happens:

“If you don’t really care [about the SRC], I think you just come here, see your patient, write your note, and then you leave. You don’t care about: did the patient get their meds, did the room get cleaned” (student)

The fact that the clinic is both mandatory and ungraded has implications, as noted by staff at the clinic:

“It’s like nobody’s really evaluating you when you’re here, you’re just here… I think having students be held more accountable for what happens here (may be helpful).” (staff member)

In this SRC, students and staff felt that the educational experience relies on self-directed learning.

The structure of the SRC provides an opportunity for students to develop leadership skills:

“It’s an opportunity for those students who are interested broadly in primary care, public health, health equity lenses, even healthcare administration, to kind of flex their interests, especially as [MCASOM SRC] leaders, but even as students within the groups to take an idea and move it to implementation or different phases of practice-based quality improvement, for example” (SRC medical director)

Students participated in the SRC in groups, and each group was led by a volunteer student leader. When asked why they volunteered to be leaders, students expressed a desire to build their leadership skills based on past experience and career goals:

“I’ve always seen myself interested in the administrative side of medicine and leading teams, and that was a little bit of what I did before I came to medical school, so I was interested in…having kind of a leadership role and working with folks to try and make that work.” (student leader)

Student leaders found the role of a peer leader challenging. All second year students started staffing the clinic at the same time, so the leaders responsible for organizing the clinic day were also experiencing the clinic for the first time and seeing their first clinic patients. The tension between being a peer and a leader pushed students to find their own leadership style and balance responsibilities with classmate expectations:

“So I just had to be really careful about how I approached things … and still straddle this line between I’m not trying to…boss everyone around, but also get everyone involved.” (student leader)

“[it would have] been beneficial to have a little bit more formality and structure to the [MCASOM SRC] leader role, but it’s also nice to have that flexibility to kind of develop your own leadership skills and be able to work with your group, what best fits with them, so just trying to find a little bit better balance” (student leader)

### SRCs as an opportunity to understand how patients experience health and care

#### Exposure to social determinants of health and barriers to healthcare access

The majority of students surveyed felt that the SRC met its stated learning objective of teaching students about SDH and barriers to healthcare access. Conversely, the student focus groups revealed that some students felt that the current clinic workflow did not leave enough room to fully explore these objectives:

“There’s no time carved out to learn about the social determinants of that individual patient. So you’d have to fundamentally change the way the clinic works if that was the goal of the student to take away that information” (student)

Students noted that their staff preceptors played a central role in starting conversations about SDH and barriers to health care access:

“Multiple occasions where I’ve seen a patient and then staffed with the physician, we’ve talked about the possibilities of, if we were in a different situation, if we were with a patient who had insurance, or if we were at the Mayo Clinic right now, how we would manage this differently.” (student)

The contradiction between the two examples above demonstrates that SDH learning opportunities were inconsistent. Students specified that the level of this focus was preceptor-dependent, which is the likely cause of this heterogeneity in student opinions. Faculty and students agreed that the SRC was uniquely positioned to address SDH in the greater curriculum:

“it’s the only place in the preclinical blocks directly putting a face to a name in terms of conceptualizations relative to social determinants of health and health care (noun).” (SRC medical director)

“I think one very valuable part of our second year is the juxtaposition between our mornings at [MCASOM SRC] and time that we spend at other buildings at Mayo, and I think that [MCASOM SRC] can be very valuable if you come in trying to cultivate an understanding of social determinants of health” (student)

While both students and faculty preceptors agreed that students were exposed to SDH and barriers to healthcare access, students in the focus groups pointed to the difference between exposure to patients with barriers to healthcare access and actively learning about these concepts.

### Patients as teachers

Students were asked about the interplay between education and quality of care, and the students’ perceived tension between learning and quality acute care:

“I think one thing that I felt kind of conflicted [about], and this is in general with student-run clinics not with [MCASOM SRC] in particular, [is] the idea of having disadvantaged patients and then using them as practice for medical students” (student)

Students acknowledged that the free service is often vital, but the fact that patients are required to see medical students was a source of discomfort. Students did feel that the quality of their contribution to patient care improved throughout the course of the year as they learned more. In contrast to the student view, interviewed patients embraced the process of student learning and did not feel like it compromised care. They described a sense of pride about being the teachers of students through allowing students to care for them:

“I would like to… give the students… a place to grow up because they are a student now and in the future they are going to be a doctor” (patient)

“*Gracias a nuestras enfermedades, ellos crecen”* [Thanks to our illnesses, they grow] (patient)

None of the three patients interviewed felt like they had worse care when asked to compare student care to care they received from physicians:

“I really couldn’t tell the difference [between seeing doctors and medical students]” (patient)

Patients enjoyed seeing students. When asked if they would prefer to just see a physician, they indicated that they would still like to see the student-physician team.

### Effect on future practice

While participation in the SRC did not have a clear impact on specialty choice, our focus groups highlighted that it affected how students think about service. It exposed students to underserved medicine, which could become an aspect of their careers regardless of their future specialty:

“At the very least, I think it exposed me to the idea that later on as a physician if I happen to go into one of these primary care specialties that I can volunteer my time in such clinics.” (student)

Students can schedule follow-up appointments with the same patient, which influenced their interest in careers with opportunities for longitudinal care:

“One thing it taught me is that I really enjoy if I can see the same patient again, so hopefully I pick a career where I have continuity with patients” (student)

## Discussion

This study reports an in-depth, mixed-methods evaluation of an SRC curriculum from multiple stakeholder perspectives. The SRC was perceived as a vital community service by all stakeholders and learning objectives were largely met. Several lessons were learned that may be relevant to other SRCs in the United States.

Students felt that the clinic was a place to develop comfort with realistic patient encounters in their pre-clinical years. These encounters involve taking a focused history, performing a physical examination, writing a note, and staffing with the physician within time constraints. This reinforces the value of SRCs to foster autonomy among medical students described in previous research [4, 19]. However, with autonomy comes a need for self-directed learning, and we found that students demonstrated varying degrees of commitment to this process. This may be related to the fact that the SRC is a mandatory component of the curriculum, thereby including some students who are less engaged in the SRC mission than others. Furthermore, the pass/fail evaluation of the course may lead to a lower value placed on its presence in the curriculum. If the SRC was a voluntary experience, which is typical of SRC experiences nationally, students who volunteered may be more committed to the process. On the other hand, the mandatory experience provides a more consistent service for patients and exposure for all medical students to an important safety net clinical setting.

The SRC is early in the students’ careers, which means that students may be underprepared to see patients, which is supported by the tension that students expressed particularly early on in the year. Despite this perceived tension on the part of students, patients perceived high quality of care. This is consistent with existing literature that suggests high quality of care at SRCs [12–14].

Importantly, patients in our study were excited about the opportunity to participate in medical student education. Given the small patient sample size in this study, the generalizability of our findings is limited, and thus this construct requires future study. The concept of patients taking a premeditated role as teachers of medical students may integrate with a participatory medicine framework that is well suited to free clinic environments, where patients and healthcare providers work together as partners [20–22].

The study identified opportunities for improvement within the SRC learning environment. In particular, students stated that exposure to patients at the SRC was not always sufficient to understand how SDH interact with healthcare delivery. Additionally, students reported varying degrees of learning based on the preceptor. This underlines the importance of strong role models to teach SDH. Students received formal curriculum in SDH before their SRC experience, which has been found in prior research to be insufficient for translating knowledge to practice [23]. This disconnect may extend to clinical experiences, which underscores the importance of “point of care” teaching about these social factors in practice. Patient-based SDH teaching is a skill set that may require intentional faculty development [24].

Previous research has demonstrated two important critiques of SRC experiences. First, a harmful hidden curriculum of stereotype reinforcement among students may emerge through SRC experiences. Most medical students were raised in families with relatively high socioeconomic position [25], which may further this divide between student and patient experiences. This speaks to the importance of faculty development and dedicated time for reflection [26]. Our findings suggest that patients may be important partners in this reflection process. Second, SRCs run the risk of providing a passive “tourist” experience of patients struggling with poverty and health problems [27]. Our findings of the tension between exposure to patients’ social conditions versus true understanding of these contexts re-enforces this concern. However, the fact that students embraced the tension more comfortably as the year went on speaks to the potential mitigating effect of a longitudinal, year-long experience. Finally, we found that some students were uneasy with the fact that SRCs needed to exist. This highlights the opportunity to teach about structural inequities in health and society that have led to a patchwork healthcare system in the United States that requires SRCs and other safety net clinics to (inadequately) address healthcare inequities [28]. These experiences may raise awareness among all medical students while positively influencing a subset of students to affect real upstream change in their careers [27].

This investigation has several strengths and limitations. The mixed-methods comprehensive evaluation is an important strength, as the quantitative and qualitative data investigated the same aims, and could be compared. The qualitative data represented several unique voices – students, staff, patients, and other stakeholders. However, survey data were only obtained from one stakeholder group (students), and it is conceivable that qualitative data did not achieve saturation across the stakeholder groups. Finally, the focus on a single SRC may not be generalizable to other SRCs.

## Conclusions and recommendations

SRCs are a setting where quality patient care can be delivered while achieving key learning objectives: participation in a real-world clinic, exposure to SDH and barriers to healthcare access, and opportunities for student leadership. The longitudinal consistency of the applied curriculum may contribute to meeting these objectives. The curricular experience should be supplemented with faculty development for real-time teaching about SDH in practice and by intentional student reflections on the structural social contexts that disproportionately affect SRC patients. Future research may also focus on the role of patients as teachers in SRCs.

## Supplementary Information

**Additional file 1:.** Student survey; Survey distributed to students via email.

**Additional file 2:.** Interview and focus group guides; The interview and focus group guides that investigators used for qualitative data acquisition.

## Data Availability

All data generated or analysed during this study are included in this published article [and its supplementary information files].
