# Supplementary material for: A mixed methods study to evaluate the impact of a student-run clinic on undergraduate medical education
Source: BMC Med Educ. 2021 Mar 25;21:182. doi: 10.1186/s12909-021-02621-y (PMC7992336; doi:10.1186/s12909-021-02621-y)
Supplement: Supplementary file 1 — Additional file 1:. Student survey; Survey distributed to students via email. [file 12909_2021_2621_MOESM1_ESM.docx]

**Additional File 1: Student Survey**

1. What year are you in medical school (or in the case of gap years, what was the last year you completed)?
   1. M1
   2. M2
   3. M3
   4. M4
2. Gender
   1. Male
   2. Female
   3. Prefer not to say
   4. Other
3. What is the most likely specialty category you will go into?
   1. Primary Care (family medicine, internal medicine, OB GYN, pediatrics, geriatrics)
   2. Internal medicine subspecialty
   3. Pediatric subspecialty
   4. Surgical specialty
   5. Other

This survey is a part of an effort to evaluate the REACH Clinic on how well it is fulfilling its stated objectives. Please mark on the scale the degree to which you agree or disagree with the below statements.

1. REACH provides a vital community service.
   1. Strongly Agree
   2. Agree
   3. Neutral
   4. Disagree
   5. Strongly Disagree
2. REACH allowed me to explore and better understand social determinants of health.
   1. Strongly Agree
   2. Agree
   3. Neutral
   4. Disagree
   5. Strongly Disagree
3. REACH allowed me to explore and better understand barriers to healthcare access.
   1. Strongly Agree
   2. Agree
   3. Neutral
   4. Disagree
   5. Strongly Disagree
4. REACH serves as an opportunity to practice patient centered medical evaluation and examination.
   1. Strongly Agree
   2. Agree
   3. Neutral
   4. Disagree
   5. Strongly Disagree
