# Supplementary material for: A mixed methods study to evaluate the impact of a student-run clinic on undergraduate medical education
Source: BMC Med Educ. 2021 Mar 25;21:182. doi: 10.1186/s12909-021-02621-y (PMC7992336; doi:10.1186/s12909-021-02621-y)
Supplement: Supplementary file 2 — Additional file 2:. Interview and focus group guides; The interview and focus group guides that investigators used for qualitative data acquisition. [file 12909_2021_2621_MOESM2_ESM.docx]

**Additional File 2: Interview and focus group guides**

**Patient Interview**

1. How did you hear about the SRC?
2. We’d like to hear about the things that patients like about the SRC and the things that patients would like to see improved. What are some of the things that you like about the SRC?
3. How about things the clinic could be doing better? What recommendations do you have to improve the SRC?
4. The clinic is staffed by medical students who are currently in training. How would you describe your experience being seen by a medical student at the SRC?
   1. What are some of the benefits you have experienced when seeing a medical student?
   2. What are some of the challenges you have experienced when seeing a medical student?
5. The clinic exists to help patients in the community, but there is also a goal to give the medical students experience working with patients in a community setting—not just within the Mayo Clinic buildings. What do you think of the goal to give students that experience?
6. Is the SRC set up to help students learn about patients’ experiences with health and health care?
7. What would you like the students to learn about how to best provide care for people in the community?

**Staff Focus Group**

1. Tell me about your role with SRC.
   1. How did you get involved?
   2. What makes you want to be involved with a program like this?
2. We’d like to better understand how the program is operating to provide a vital service for community members/patients and for the students who staff the clinic. Let’s start with service to the community. How do you think the clinic is doing in terms of its objective to provide a vital community service for patients?
   1. Who do you view as the main intended beneficiaries of SRC?
      1. How are we doing in terms of reaching those people?
   2. How do you view SRC’s role in the broader Rochester community?
   3. How would you describe support for the SRC?
      1. What resources does SRC need to more effectively operate, if any?
3. One of the stated learning objectives for students at SRC is to explore the social determinants of health through the lives and circumstances of REACH patients. To what extent do you think SRC is meeting this objective?
   1. In what ways is the program designed to do this well? Can you share examples of this?
   2. What are some of the challenges, if any, to meeting this objective for students?
   3. What changes do you see in students over the course of their year staffing SRC?
4. Another stated learning objective is for students to gain understanding of the barriers to healthcare access. To what extent do you think SRC is meeting this objective?
   1. In what ways is the program designed to do this well? Can you share examples of this?
   2. What are some of the challenges, if any, to meeting this objective for students?
5. Finally, SRC is designed to help students practice patient-centered medical evaluation and examinations. How do you think this goal is being met?
   1. Are there ways to improve the student experience?
   2. How about ways to improve the patient experience of working with the students?
6. I asked earlier about support for the SRC. Thinking more specifically about the objectives related to student learning, how would you describe support (in the clinic, in the organization, in the community)?
7. Do you have any other recommendations to improve SRC (for patients, the community, and student learners)? Is there anything else we should know about how or why it is or is not successful?

**Stakeholder Interview**

1.    Tell me about your role with the SRC.

a.    How did you get involved?

b.    What makes you want to be involved with a program like this?

2.    We’d like to better understand how the program is operating to provide a vital service for community members/patients and for the students who staff the clinic. Let’s start with service to the community. How do you think the clinic is doing in terms of its objective to provide a vital community service for patients?

a.    How do you know whether the program is achieving its aims in terms of community benefit?

b.    Who do you view as the main intended beneficiaries of the SRC?

               i. How are we doing in terms of reaching those people?

c.    How do you view SRC’s role in the broader Rochester community?

d.    How would you describe support for the SRC?

               i. What resources does SRC need to more effectively operate, if any?

3.    What do you see as the program’s objectives related to medical students’ participation in the SRC?

4.    One of the stated learning objectives for students at SRC is to explore the social determinants of health through the lives and circumstances of REACH patients. To what extent do you think SRC is meeting this objective?

a.    In what ways is the program designed to do this well? Can you share examples of this?

b.    What are some of the challenges, if any, to meeting this objective for students?

5.    Another stated learning objective is for students to gain understanding of the barriers to healthcare access. To what extent do you think SRC is meeting this objective?

a.    In what ways is the program designed to do this well? Can you share examples of this?

b.    What are some of the challenges, if any, to meeting this objective for students?

6.    How do you know whether the program is achieving its aims in terms of student learning?

7.    Are there ways to improve the student experience?

a.    How about ways to improve the patient experience of working with the students?

8.    I asked earlier about support for the SRC. Thinking more specifically about the objectives related to student learning, how would you describe support (in the clinic, in the organization, in the community)?

9.    Do you have any other recommendations to improve SRC (for patients, the community, and student learners)? Is there anything else we should know about how or why it is or is not successful?

**Student Focus Group**

1. One of the stated learning objectives for students at SRC is to explore the social determinants of health through the lives and circumstances of REACH patients. To what extent do you think SRC is meeting this objective?
   1. In what ways is the program designed to do this well? Can you share examples of this?
   2. What are some of the challenges, if any, to meeting this objective?
2. Another stated learning objective is for students to gain understanding of the barriers to healthcare access. To what extent do you think SRC is meeting this objective?
   1. In what ways is the program designed to do this well? Can you share examples of this?
   2. What are some of the challenges, if any, to meeting this objective?
3. Finally, SRC is designed to help students practice patient-centered medical evaluation and examinations. How do you think this goal is being met?
4. How do you think SRC compares to other clinical education experiences in the first two years of medical school (Pediatrics, Advanced Doctoring etc.)?
5. How has SRC had an impact on your ideas about your goals for a career in medicine?
   1. Has it affected specialty choice?
   2. Has it affected in what settings you might want to practice?
6. Do you have any other recommendations to improve SRC (for patients, the community, and student learners)? Is there anything else we should know about how or why it is or is not successful?

**SRC Student Leaders Focus Group**

1. Tell me about your role as a reach leader.
   1. Why did you get involved?
   2. What makes you want to be involved as a leader?
2. One of the stated learning objectives for students at SRC is to explore the social determinants of health through the lives and circumstances of REACH patients. To what extent do you think SRC is meeting this objective?
   1. In what ways is the program designed to do this well? Can you share examples of this?
   2. What are some of the challenges, if any, to meeting this objective?
3. Another stated learning objective is for students to gain understanding of the barriers to healthcare access. To what extent do you think SRC is meeting this objective?
   1. In what ways is the program designed to do this well? Can you share examples of this?
   2. What are some of the challenges, if any, to meeting this objective?
4. Finally, SRC is designed to help students practice patient-centered medical evaluation and examinations. How do you think this goal is being met?
5. How do you think SRC compares to other clinical education experiences in the first two years of medical school (Pediatrics, Advanced Doctoring etc.)?
6. How has SRC had an impact on your ideas about your goals for a career in medicine?
   1. Has it affected specialty choice?
   2. Has it affected in what settings you might want to practice?
7. Do you have any other recommendations to improve SRC (for patients, the community, and student learners)? Is there anything else we should know about how or why it is or is not successful?
